# Distal and proximal family predictors of adolescents' smoking initiation and development: A longitudinal latent curve model analysis

**DOI:** 10.1186/1471-2458-11-911

**Published:** 2011-12-09

**Authors:** Tore Tjora, Jørn Hetland, Leif Edvard Aarø, Simon Øverland

**Affiliations:** 1 Uni Health, Uni Research, Christiesgate 12, 5015 Bergen, Norway; 2 Department of Psychosocial Science, Faculty of Psychology, University of Bergen, Christiesgate 13, 5015 Bergen, Norway; 3 Division of Mental Health, National Institute of Public Health, Sandakerveien 24 C, 0473 Oslo, Norway; 4 Department of Health Promotion and Development, Faculty of Psychology, University of Bergen, Christiesgate 12, 5015 Bergen, Norway

## Abstract

**Background:**

Studies on adolescent smoking indicate that the smoking behaviours of their parents, siblings and friends are significant micro-level predictors. Parents' socioeconomic status (SES) is an important macro-level predictor. We examined the longitudinal relationships between these predictors and the initiation and development of adolescents' smoking behaviour in Norway.

**Methods:**

We employed data from *The Norwegian Longitudinal Health Behaviour Study (NLHB)*, in which participants were followed from the age of 13 to 30. We analysed data from the first 5 waves, covering the age span from 13 to 18, with latent curve modeling (LCM).

**Results:**

Smoking rates increased from 3% to 31% from age 13 to age 18. Participants' smoking was strongly associated with their best friends' smoking. Parental SES, parents' smoking and older siblings' smoking predicted adolescents' initial level of smoking. Furthermore, the same variables predicted the development of smoking behaviour from age 13 to 18. Parents' and siblings' smoking behaviours acted as mediators of parents' SES on the smoking habits of adolescents.

**Conclusions:**

Parents' SES was significantly associated, directly and indirectly, with both smoking initiation and development. Parental and older siblings' smoking behaviours were positively associated with both initiation and development of smoking behaviour in adolescents. There were no significant gender differences in these associations.

## Background

Among adolescents in the US and elsewhere, smoking rates have declined [1,2]. However, despite a general decline over cohorts and the well-documented and well-disseminated fact that smoking has severe health consequences, many adolescents still start smoking.

It therefore remains an important question what leads young non-smokers to take up smoking. Candidate factors such as the smoking behaviours of parents, siblings and friends are positively correlated with adolescents' smoking [3-6]. Micro-level (proximal) factors such as perceived social pressure from peers, parents and siblings regarding smoking can form subjective norms that may either increase or reduce the chance that adolescents will start smoking. Smoking may also be influenced directly or indirectly by macro-level (distal) factors such as parents' socioeconomic status (SES) [7].

For adult populations, the association between SES and smoking is well established [8-10]: people with a lower SES are more likely to smoke [8] and to start earlier [8], whereas smokers with a high SES are more likely to quit smoking [11]. It is less certain to what extent parents' SES is associated with adolescents' smoking behaviour. A number of studies have demonstrated associations between parents' SES and adolescents' general health behaviours [9,12,13] and between adolescents' health behaviours and the SES they later acquire [14,15]. For smoking, in particular, despite mixed results, a recent systematic review found that most of the evidence points towards a higher risk of smoking among adolescents with a lower SES [16]. However, little is known about the relative importance of SES for adolescent smoking initiation and development.

It is likely that part of the possible association between parents' SES and adolescent smoking behaviour is mediated through micro-level factors, both through subjective norms and through smoking habits of parents. If parents [3,17-24] and/or older siblings smoke, adolescents are more likely to initiate smoking [4]. Whether an adolescent is permitted by parents to smoke predicts smoking initiation and maintenance [17,18,25,26] and even predicts his/her preferences for future smoke-free living quarters [27]. Adolescents from non-smoking homes are thus less likely to start smoking, and banning smoking from one's home reduces the risk that adolescents will begin smoking, even when parents smoke [18] although a recent review found that parents' smoking appears to reduce the effect of a household smoking ban [26].

Having friends who smoke is a major correlate of smoking initiation [3,24,28-32]. Whereas the previously mentioned predictors, such as parents' smoking behaviour, older siblings' smoking behaviour and parents' SES, are less likely to change as a result of adolescents' smoking behaviour, there may be mutual processes of influence between adolescents own smoking habits and those of their friends. Adolescent smokers more often befriend others who smoke [33], and adolescent smoking trajectories are formed both by social influences and by social selection [5,30,33-35]. Because adolescents' own smoking behaviour and their friends' smoking status develop side-by-side, using smoking among friends to predict own smoking behaviour may be a statistical oversimplification and may lead to an underestimation of the relative importance of other predictors.

In this article, we examined smoking initiation and development using data from a longitudinal study covering the important age span of 13-18 years. Specifically, we wanted to investigate the relative importance of friends' smoking behaviour, parental smoking behaviour, older siblings' smoking behaviour and parents' SES as predictors of adolescents' smoking habits at baseline and over time with latent growth curve modelling. Two main hypotheses were tested: 1) adolescents' smoking behaviour is positively associated with parental, older siblings' and friends' smoking behaviours and 2) parents' SES is both directly and indirectly associated with smoking initiation and development. To avoid statistical overcontrol, models were tested also without the respondents' best friends' smoking behaviour as a predictor.

## Methods

In this study, we employed data from *The Norwegian Longitudinal Health Behaviour Study (NLHB) *[36]. This dataset is used by the research-group that collected and owns the data, it is not publicly available. This is a longitudinal study of perceived health and health-related behaviour, in which a cohort of adolescents who were 13 years old in 1990 completed questionnaires from nine consecutive surveys between 1990 and 2007 (ages 13-30). The participants' parents completed one questionnaire in 1990. A representative sample from Hordaland, a county in western Norway, was selected. A total of 1,195 13-year-olds and their parents were invited to participate, of which 924 initially participated. All participants were invited to participate at later stages, which brought the total number of participants available for this analysis to 1053. In the present study, we focus on smoking initiation processes in adolescence. Hence, we use the first five waves in 1990, 1991, 1992, 1993 and 1995 (ages 13-18).

### Variables

Smoking was measured by the same item in all waves: "How often do you smoke?" ("Every day", "every week", "at least once a week", "not at all.") This categorical variable of smoking frequency was used in the growth models and to construct two dichotomous variables of smoking: a) those smoking every day versus all others and b) those smoking every day or every week versus all others. Friends' smoking behaviour was measured in 1990, 1992 and 1995 by two questions: 1) "How many of your best friends smoke?" ("None," "Less than half of my friends," "About half of my friends," "More than half of my friends" and "Almost all my friends") and 2) "Does your best friend smoke?" ("Yes" or "No"). Regarding friends' smoking the first wave was used in the latent growth models and all three were used in the descriptive analyses. For parents' smoking behaviour, we used information from the first wave only. Here, one item inquired about whether each of the parents smoked daily (coded 2), occasionally (coded 1) or did not smoke at all (coded 0). The two items on parental smoking were added to construct a parents' smoking scale. Sibling smoking behaviour was identified through a dichotomous question in the first wave: "Do you have older siblings who smoke?," with response categories "Yes" and "No." The participants were also asked whether they were permitted to smoke by their parents: "Are you allowed to smoke at home?" This is a direct translation of the Norwegian wording; a more appropriate translation, carrying the same meaning as the Norwegian expression, would be: "Do your parents allow you to smoke?"

The adolescents were asked about each of their parents' level of education in the third wave. "Elementary (7 years)" and "Secondary (9 years)" were coded 0, "Upper secondary (vocational and general)" was coded 1, "University/higher education" was coded 2 and "Other education" and "He/she is deceased" were coded missing. Parents were asked about their occupation in the first wave (1990) and were given examples of the fixed answer categories in four groups: "Not working," "Unskilled manual" and "Skilled manual" (all coded 0); "Service 1," "Service 2," "Office," "Farming, fishing" and "Own business" (all coded 1); and "Lower Official" and "Higher Official" (all coded 2). "Unidentified" and "No answer" were coded missing. As the number of parents in the family varies, we used the sum of the mean levels of education and job to construct an ordinal scale reflecting parents' SES. Participants scoring in the lower 25% of this SES scale were coded "low SES," the next 50% were coded "mid SES" and the higher 25% were coded "high SES" to construct a categorical variable of parents' SES. We used this variable to construct two dichotomous variables: a) High SES versus low and mid (labeled "high SES") and b) low SES versus mid and high (labelled "low SES").

### Statistical analysis

Latent Curve Modeling (LCM) with Mplus version 5.21 was used to examine developmental trajectories in smoking across the five waves. Descriptive analyses were performed in SPSS 15.0 for Windows. For the descriptive statistics and the construction of the SES variables, we used pairwise deletion of missing data. In Mplus, the default WLSMV estimation was used because the smoking variable was categorical [37]. We used RMSEA and CFI to evaluate the goodness of fit for the tested models. Both CFI and RMSEA are measures that indicate the degree to which a model fits with the data. The closer CFI is to one [38] and the closer RMSEA is to 0, the better the model fits [38]. Parents and older siblings' smoking behaviours were measured at baseline only. For internal consistency, the best friend's smoking behaviour was used at baseline only in the growth model.

### Latent growth curves

The strategy for statistical analyses was to first establish whether smoking could be studied using an LCM approach and then to investigate to what degree our various predictors were associated with the initiation and development of smoking. Technically, we did this by first estimating an unconditional linear univariate LCM model of adolescent smoking behaviour to estimate the best-fitting growth function over time, M1 (Table 1). We controlled that both retained latent variables (intercept and slope) had significant variance.

**Table 1:** Fit indices for the tested models

Model	n	χ^2^	df	CFI	RMSEA
M1: Univariate LCM	1052	46.73	9	0.99	0.063

M2: Conditional LCM: Time invariant covariates	1053	62.75	18	0.99	0.049

M3: M2 without insignificant paths (Figure 1)	1053	62.83	19	0.99	0.047

M4: M3 without friend's, with mediators (Figure 2)	1053	68.43	17	0.99	0.054

We then regressed the latent variables that reflected the initial level (intercept) and the growth factor (slope factor) on gender, the best friend's smoking behaviour, parents and older siblings' smoking behaviours, and parents' SES (M2, Table 1). Initial analysis showed that whether an adolescent is allowed to smoke was very infrequent and the distribution was skewed, it was therefore excluded from the growth models. We used the dichotomous "high SES" because this variable was significantly associated with smoking at baseline, whereas "low SES" was not. We then excluded the best friend's smoking behaviour from the model due to the possible reciprocal influence with the participant's own smoking behaviour (M3, Table 1) and its potentially strong effects on other associations in the model. We used Sobel's test to examine whether parents' SES had an indirect association with the growth curve in the final model.

## Results

The rate of daily smoking increased from 3% at age 13 (1990) to 31% at age 18 (1995), which is equivalent to an average increase of 5.6 percentage points per year (Table 2). The increase in smoking "at least once a week" and "smoking every week" was much weaker. There was a significant increase in smoking rates between each wave and the subsequent one. The reported rate for the best friend being a smoker also increased significantly, from 11% to 48%, corresponding to an annual growth exceeding 7 percentage points (Table 2). Furthermore, the proportion of participants who reported that none of their friends were smokers decreased significantly, from 73% when the participants were 13 to 11% when they were 18 (Table 2).

**Table 2:** Prevalence in % (n) of own and friends' smoking behaviour

	1990	1991	1992	1993	1995
Smoking status		χ^2 ^= 388.77, df = 9*	χ^2 ^= 406.00, df = 9*	χ^2 ^= 398.55, df = 9*	χ^2 ^= 359.20, df = 9*

Non-smoker	88 (781)	76 (717)	65 (590)	66 (463)	52 (404)

LT 1 time/week	7 (63)	12 (111)	12 (111)	8 (55)	11 (83)

Every week	2 (14)	4 (39)	6 (54)	5 (33)	6 (48)

Every day	3 (29)	9 (82)	18 (160)	22 (154)	31 (241)

NA	(354)	(292)	(326)	(536)	(465)

Best friend's smoking status		χ^2 ^= 84.74, df = 1*		χ^2 ^= 78.48, df = 1*	

Yes	11 (94)		34 (304)		48 (372)

No	89 (785)		66 (592)		52 (399)

NA	(362)		(345)		(470)

Friends' smoking status		χ^2 ^= 123.62, df = 16*		χ^2 ^= 169.95, df = 16*	

None	73 (646)		34 (308)		11 (83)

Less than half	20 (177)		35 (316)		36 (282)

About half	3 (24)		15 (135)		21 (163)

More than half	1 (12)		7 (62)		15 (115)

Almost all	3 (23)		10 (91)		17 (134)

NA	(359)		(329)		(464)

*Significant (*p *< 0.001)

LT = at least

NA = not applicable

There was no gender difference in smoking status (no smoking versus smoking at all) at baseline (χ^2 ^= 0.04, df = 1, *p *> 0.05) (Table 3). Both father's (χ^2 ^= 5.74, df = 1, *p *< 0.01) and mother's smoking behaviours (χ^2 ^= 7.86, df = 1, *p *< 0.01) were positively associated with adolescents' smoking behaviour (Table 3). The distribution of whether the adolescent was allowed by their parents to smoke was too skewed to permit chi-square testing (expected frequency < 5). Parents' high SES was significantly associated with lower smoking prevalence (χ^2 ^= 5.93, df = 2, *p *< 0.05) (Table 3). However, the two strongest predictors for adolescents to smoke were whether older siblings were smokers (χ^2 ^= 51.43, df = 1, *p *< 0.001) and, in particular, whether the best friends were smokers (χ^2 ^= 305.36, df = 1, *p *< 0.001) (Table 3).

**Table 3:** Smoking at all versus no smoking across predictors at baseline (1990) and final wave (1995)

		Own smoking behaviour 1990				Own smoking behaviour 1995			
	
		Yes	No	n	Difference^a^	Yes	No	n	Difference^a^
Gender	Boy	12%	88%	887	χ^2 ^= .04, df = 1, *p *> 0.05	45%	55%	776	χ^2 ^= 2.58, df = 1, *p *> 0.05
	
	Girl	12%	88%			51%	49%		

Father smoking	Yes	15%	85%	858	χ^2 ^= 5.74, df = 1, *p *< 0.01	59%	41%	617	χ^2 ^= 24.91, df = 1, *p *< 0.05
	
	No	9%	91%			39%	61%		

Mother smoking	Yes	15%	85%	874	χ^2 ^= 7.86, df = 1, *p *< 0.01	58%	42%	631	χ^2 ^= 16.75, df = 1, *p *< 0.05
	
	No	9%	91%			41%	59%		

Older sibling smoking	Yes	27%	73%	846	χ^2 ^= 51.43, df = 1, *p *< 0.001	66%	34%	610	χ^2 ^= 18.93, df = 1, *p *< 0.00
	
	No	7%	93%			44%	56%		

Best friend smoking	Yes	67%	33%	874	χ^2 ^= 305.36, df = 1, *p *< 0.00	78%	22%	631	χ^2 ^= 25.59, df = 1, *p *< 0.00
	
	No	5%	95%			45%	55%		

Allowed smoking	Yes	33%	67%	764	NA^b^	33%	67%	556	NA^b^
	
	No	11%	89%			47%	53%		

Parents' SES	Low	12%	88%	431	χ^2 ^= 5.93, df = 2, *p *< 0.05	49%	51%	351	χ^2 ^= 1.05, df = 2, *p *> 0.05
	
	Mid	11%	89%			46%	54%		
	
	High	4%	96%			52%	48%		

^a^Chi-square test of difference

^b^Not estimable because expected frequency was lower than 5

Smoking prevalence increased among both boys and girls from one data collection to the next over the entire 1990-1995 period (data not shown). The gender differences were mixed over the follow-up period; girls generally had a higher smoking prevalence, but the shape of the gender differences varied both as a function of how smoking is defined and depending on which of the waves were emphasised (data not shown).

### Dropout

The attrition rate from the baseline to wave five was 36%. Neither the participant's own smoking behaviour nor the best friend's smoking behaviour was significantly associated with attrition. However, participants with a high SES had a significantly lower attrition rate than those with a low and mid SES. In a post-hoc analysis regressing the intercept and slope of the smoking growth curve on whether the participants had a valid SES value, a missing SES was positively associated with intercept but was not significantly associated with slope.

### Univariate unconditional growth model of adolescents' smoking behaviour

The univariate model (Table 1, M1) had a fair to good fit (χ^2 ^= 46.73(9), CFI = 0.99, RMSEA = 0.06). The intercept mean is set to 0 by Mplus when the outcome variables are categorical, and the significant slope mean of .32 (*p *< 0.05) represents an increasing developmental trend of smoking behaviour with increasing age. Furthermore, the intercept $\left({\overset{⌢}{\sigma }}_{\alpha }^{2}=.91,\text{p}\;\text{ < }\;\text{.05,}\;\text{S}.\text{E}.=.05\right)$ and slope $\left({\overset{⌢}{\sigma }}_{\beta }^{2}=.20,\text{p}\;\text{ < }\;\text{.05,}\;\text{S}\text{.E}.=.05\right)$ were characterised by significant variances indicating individual variability of both starting point and slope of smoking behaviour. There was a significant correlation between intercept and slope (0.35, *p *< 0.05).

### Univariate conditional growth model, best friend's smoking behaviour, parent's SES and smoking behaviour at home (Figure 1)

**Figure 1:**
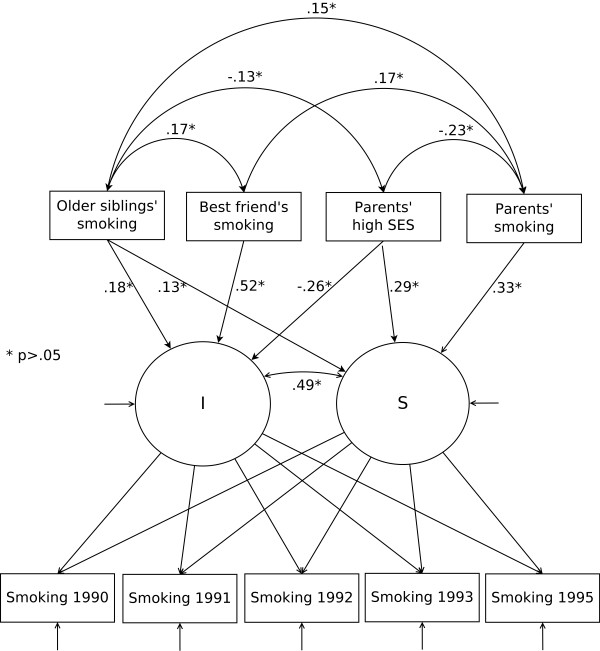
**Own smoking by best friend's smoking, parents' and older siblings' smoking, and parents' SES**. A conditional univariate latent curve model covering the age span from 13 to 18

The question whether smoking was allowed by parents was excluded because very few informants were allowed to smoke. The unconditional LCM, reflecting the adolescents' smoking trajectory, was regressed on the best friend's smoking behaviour, parental and older siblings' smoking behaviours and parents' SES (Table 1, M2). The insignificant loadings on both intercept and slope were omitted from the model (Figure 1, Table 1, M3). All predictors had a significant impact on intercept; friends' smoking behaviour (.52, *p *< 0.05) was the strongest, followed by parents' high SES (-0.26, *p *< 0.05) and older siblings' smoking behaviour (0.18, *p *< 0.05). The best friend's smoking behaviour was the only predictor not associated with the slope. Parents' smoking behaviour was the strongest (0.33, *p *< 0.05), followed by parents' SES (0.29, *p *< 0.05) and older siblings' smoking behaviour (0.13, *p *< 0.05). Parents' SES changed from a negative association to a positive association.

### Univariate conditional growth model, parent's SES mediated through smoking behaviour at home (Figure 2)

**Figure 2:**
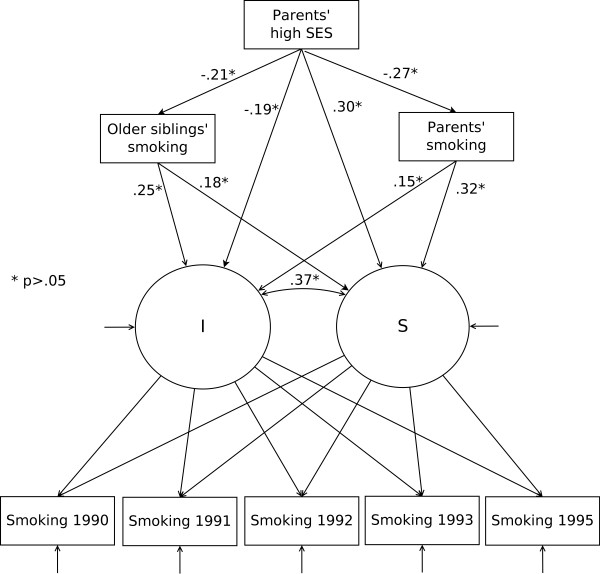
**Own smoking by parents' and older siblings' smoking and parents' socioeconomic status**. A conditional univariate latent curve model covering the age span from 13 to 18, direct and indirect associations

The unconditional LCM was also regressed on parental and older siblings' smoking behaviours and parents' SES (Table 1, M4) without the best friend's smoking behaviour. The insignificant loadings on both the intercept and slope were omitted from the model, and the final model (Table 1, M4) also included the indirect associations [39]. These indirect effects were associations between parents' high SES and the intercept and slope via both parents' smoking behaviour and older siblings' smoking behaviour (Figure 2). The final model showed good fit (χ^2 ^= 68.43, CFI = .99, RMSEA = 0.05), and all associations shown in Figure 2 were significant. Parents' high SES was significantly associated with older siblings' smoking behaviour (-0.21, *p *< 0.05), parents' smoking behaviour (-0.27, *p *< 0.05), intercept (-0.19, *p *< 0.05) and slope (0.30, *p *< 0.05). Older siblings' smoking behaviour was associated with both the intercept (0.25, *p *< 0.05) and slope (0.18, *p *< 0.05). Parents' smoking behaviour was also associated with both intercept (0.15, *p *< 0.05) and slope (0.32, *p *< 0.05).

There were also significant indirect associations. The total effect from parents' high-SES (-0.28, *p *< 0.05) on the intercept, the direct effect (-0.19, *p *< 0.05) and the indirect effects via siblings' smoking behaviour (-0.05, *p *< 0.05) and parents' smoking behaviour (-0.04, *p *< 0.05) were significant. The total effect from parents' high SES (0.18, *p *< 0.05) on the slope, the direct effect (0.30, *p *< 0.05) and the indirect effects via siblings' smoking behaviour (-0.04, *p *= 0.05) and parents' smoking behaviour (-0.08, *p *< 0.05) were also all significant.

## Discussion

### Main findings

The strong increase in adolescents' daily smoking, from 3% to 31% (Table 2) between the ages of 13 and 18, demonstrates the importance of studying predictors of smoking initiation and development during the transition between adolescence and young adulthood. Having friends who smoked was strongly associated with smoking at the initial level, and parents and siblings' smoking behaviours were positively associated with both the initial level and the development of adolescents' smoking behaviour. Parents' SES had a direct negative association with adolescents' smoking behaviour at the initial level but a direct positive association with smoking development. There were also indirect associations between parents' SES and smoking development via parents' and older siblings' behaviour, which indicates a greater importance of SES than previously indicated.

### Study strength and limitations

The main strength of this study is the longitudinal data and the inclusion of information on both the adolescents and their parents (or guardians). The initial sample of approximately one thousand adolescents was followed up five times, providing a unique possibility to monitor change over time.

There are, however, some significant limitations. The obvious limitation of missing values, to be expected in a data collection such as this, has been countered by employing the Mplus WLSMV estimator, which is useful for categorical outcome measures where missing values are allowed in observed covariates [40]. We checked whether dropout was associated with key variables and found a significant association between low parental SES and dropout, which is likely to weaken the association between parents' SES and adolescents' smoking behaviour.

Following the data collection, there has been a decline in both adolescent and adult smoking in Norway [41]. Thus, "smokers" may now have different characteristics than they did previously. A recent Danish study suggested that the social inequality in adolescent smoking has increased in the last decade [16]. This result could mean that the associations between socioeconomic status and smoking in our data are weaker than the true association. Differences between the models presented in this publication and similar models based on more recent data, however, would likely be a matter of strengths of associations rather than a fundamental and qualitative difference in the social influence processes described here.

For parent-based SES, we used parents' job status and education level, which are among the most commonly used predictors of socioeconomic variation in smoking [8,42]. However, the SES scale employed here has not been validated and was constructed and applied with face validity. Nevertheless, our finding that parental SES was associated with parental smoking behaviour was as expected [8], which strengthens our confidence in this measurement. Another weakness regarding the SES measure is that the questions on parents' level of highest completed education were asked to the participants at age 15 rather than to the parents directly. Not all questions were asked at every wave, which has prevented us from modelling the impact of changes in parents' and older siblings' smoking behaviours.

### Interpretation

The main aim of the study was to investigate the development of adolescents' smoking behaviour and its predictors over 5 years in adolescence. The development of adolescents' smoking habits may be seen as an accumulative process, a claim supported by the positive association between initial smoking behaviour and the development of smoking behaviour. This means that the earlier in life one begins smoking, the higher the risk of being a smoker also later in life, a finding also supported in other studies [43]. We found support for both hypotheses: smoking was positively associated with parents', older siblings' and friends' smoking behaviours. We also found that parents' SES was both directly and indirectly associated with smoking initiation and development.

The associations between friends' and adolescents' smoking behaviours have been thoroughly investigated [5,24,30,33,34], and friends' smoking habits stand out as more than an important predictor. However, in our study, friends' smoking behaviour did not contribute significantly to masking the associations between family predictors and smoking. As mentioned, the influence processes between adolescents' own smoking behaviour and friends' smoking behaviour are reciprocal. Therefore, we ran the last model without the best friend's smoking behaviour as a predictor.

The exclusion of the best friend's smoking behaviour in the last model should not be understood as a devaluation of peer influence. On the contrary, because the best friend's smoking behaviour is much more than simply a predictor, we preferred to present a model that omitted this factor. In the present study, there were only minor differences between the models with and without the best friend's smoking behaviour. Based on conceptual difficulties in treating the best friend's smoking behaviour as merely a predictor and the absence of major differences between the two models including or excluding the best friend's smoking behaviour, we removed the best friend's smoking behaviour as a predictor in the final model to avoid statistical over-control.

Both parental and older siblings' smoking behaviours were significantly associated with initiation levels of adolescent smoking, with older siblings' behaviour being the strongest correlate. The influence from both parents and, to some degree, older siblings may have begun long before the participants reached the age of 13. Other studies have found both parental smoking behaviour and parents' attitudes towards their children's smoking to be influential factors for adolescent smoking [17-20], and our results support the importance of parents' behaviour. The model including the best friend's smoking behaviour (Figure 1) also highlights the importance of parental and siblings' smoking behaviours through the association between the best friend's smoking behaviour and sibling and parental smoking behaviours. This finding indicates that the importance of the family goes beyond the direct associations between adolescents' smoking behaviour and family members' smoking behaviour. Further, the important factor of the best friend's smoking behaviour did not significantly predict smoking development, but both parents' and siblings' smoking behaviours did predict smoking development, highlighting the enduring importance of the family. However, the best friend's smoking behaviour was the strongest predictor of smoking initiation. Despite its ambiguous role in the causal processes and pathways leading to smoking in adolescence, the smoking behaviour of adolescents' best friends must be assumed to be important.

Parents' SES, which reflects a more distal factor [7], is also important. Parents' SES was both directly and indirectly associated with initiation levels of smoking behaviour and the development through adolescence. Like parents' smoking behaviour, parents' SES influence may begin earlier than age 13 through the association with parental and siblings' smoking behaviours and through the link to friends' smoking behaviour (Figure 1). We found that a high SES was negatively associated with parents' and older siblings' smoking behaviours. A high SES also had a direct negative association with initiation level. This finding could suggest that parental smoking behaviour is an important factor in explaining the association between adolescents' smoking behaviour and their parents' SES [44]. This interpretation is modestly supported by a previous study that demonstrated the importance of the association between parents' behaviour and adolescents' smoking behaviour [45]. Taken together, these results show the importance of incorporating both micro--and macro-level family factors in understanding and explaining adolescents' smoking initiation.

Parental smoking behaviour, siblings' smoking behaviour and parents' SES also have significant associations with adolescents' smoking development. The positive associations between parents and siblings' smoking behaviours and the development of adolescents' smoking behaviour indicate that these proximal factors may augment smoking development. A high parental SES was positively associated with smoking development. This finding, combined with the negative association between smoking initiation and parental SES, indicates that the direct impact of parents' SES is diminishing between age 13 and age 18. This finding is further supported by the lack of association between participants' smoking behaviour and parents' SES at age 18 (Table 3). However, a high parental SES contributed indirectly and negatively to smoking development via both parental and older siblings' smoking behaviours.

The influence from family, including parents and siblings, may also have practical implications. Interventions based on the impact of friends' smoking behaviour may be more effective if a broader perspective is adopted. A further recognition of the influence of families, including the families of friends, may contribute to improve interventions directed towards adolescents. The importance of parents' socioeconomic status should also be considered when planning and framing school-based interventions [46,47].

## Conclusion

This study indicates that, from a cross-sectional perspective, adolescents' smoking habits are much more strongly associated with their peers' smoking habits than with their parents or siblings' smoking habits. From a longitudinal perspective, however, it seems that parents' SES and parental and older siblings' smoking behaviours are of enduring importance for smoking among adolescents. Over time, parents' SES both directly and indirectly predicts smoking initiation and development among children. Although the direct association between parents' SES diminishes as adolescents grow older, the combination of parental and sibling influence is important. There are calls for more specific anti-smoking interventions that specifically target at-risk individuals and smokers [41,48], and our results lend support to interventions that address both distal and proximal factors.

## Competing interests

The authors declare that they have no competing interests.

## Authors' contributions

TT carried out the work for the present study in collaboration with SØ, JH and LEAA. All authors contributed to the conceptual matters, statistical analysis, interpretation and writing of the manuscript. All authors have seen and approved the final version.

## Pre-publication history

The pre-publication history for this paper can be accessed here:

http://www.biomedcentral.com/1471-2458/11/911/prepub
